# Supplementary material for: Impact of Sex on Rehospitalization Rates and Mortality of Patients with Heart Failure with Preserved Ejection Fraction: Differences Between an Analysis Stratified by Sex and a Global Analysis
Source: J Pers Med. 2025 Jul 8;15(7):297. doi: 10.3390/jpm15070297 (PMC12300905; doi:10.3390/jpm15070297)
Supplement: Supplementary file 1 [file jpm-15-00297-s001.zip › jpm-3711125-supplementary.pdf]

Supplementary Table 1. Multivariable logistic regression analysis using dichotomous outcomes (mortality and rehospitalization), stratified by sex

Table 1A. Multivariate analysis: Logistic regression analysis for all-causes of mortality for entire population and stratified by sex.

	Entire population			Men			Women		
**Predictors**	Odds Ratio	95% CI	P value	Odds Ratio	95% CI	P value	Odds Ratio	95% CI	P value
Sex (Male)	1.34	1.08 – 1.66	0.007						
Age (years) [>75]	2.86	2.24 – 3.68	<0.004	3.43	2.53 – 4.70	<0.001	2.82	2.03 – 3.97	<0.001
Body mass index ≤ 30 kg/m^2	1.49	1.22 – 1.81	<0.001	-	-	-	1.88	1.45 – 2.43	<0.001
Charlson index									
0	Ref.						Ref.		
1 – 2	1.19	0.68 – 2.19	0.556	-	-	-	1.29	0.68 – 2.63	0.455
3 – 4	1.69	0.97 – 3.12	0.075	-	-	-	2.03	1.07 – 4.15	0.038
>4	2.12	1.20 – 3.96	0.013	-	-	-	2.87	1.47 – 5.98	0.003
Loop diuretic	1.42	1.13 – 1.81	0.003	-	-	-	1.67	1.21 – 2.31	0.002
Hemoglobin (g/dl)	0.82	0.77 – 0.88	<0.001	0.78	0.72 – 0.84	<0.001	-	-	-
LVEF ≥55%	-	-	-	-	-	-	0.65	0.45 – 0.94	0.020

Table 1B. Multivariate analysis: Logistic regression analysis for heart failure rehospitalization for the entire population and stratified by sex.

	Entire population			Men			Women		
**Predictors**	Odds Ratio	95% CI	P value	Odds Ratio	95% CI	P Value	Odds Ratio	95% CI	P value
Sex (Male)	1.09	0.91 – 1.31	0.330						
Age (years) [>75]	1.18	0.97 – 1.43	0.094	1.32	0.98 – 1.78	0.066	1.18	0.90 – 1.54	0.235
Body mass index ≤ 30 kg/m2	-	-	-	0.68	0.51 – 0.91	0.008	-	-	-
Loop diuretic	1.43	1.17 – 1.76	<0.001	-	-	-	1.41	1.08 – 1.87	0.013
Hemoglobin (g/dl)	0.90	0.86 – 0.95	<0.001	-	-	-	0.84	0.78 – 0.91	<0.001
Atrial fibrillation	1.48	1.25 – 1.76	<0.001	1.33	1.00 – 1.76	0.051	1.66	1.32 – 2.09	<0.001
Coronary disease	-	-	-	1.46	1.02 – 2.08	0.036	-	-	-
